# Biological activity and interaction mechanism of the diketopiperazine derivatives as tubulin polymerization inhibitors†

**DOI:** 10.1039/c7ra12173c

**Published:** 2018-01-03

**Authors:** Zhenhua Tian, Yanyan Chu, Hui Wang, Lili Zhong, Mengyan Deng, Wenbao Li

**Affiliations:** School of Medicine and Pharmacy, Ocean University of China Qingdao 266003 China wbli92128@163.com; Innovation Center for Marine Drug Screening and Evaluation, Qingdao National Laboratory for Marine Science and Technology Qingdao 266071 China; Marine Biomedical Research Institute of Qingdao Qingdao 266071 China

## Abstract

Microtubules are a favorable target for development of anticancer agents. In this study, the anti-proliferative activities of plinabulin and six diketopiperazine derivatives were evaluated against human lung cancer cell line NCI-H460 and human pancreatic cancer cell line BxPC-3. The inhibition activities on these microtubules were assessed by tubulin polymerization and immunofluorescence assays. To gain insight into the interaction mechanism of the derivatives and tubulin, a molecular dynamics simulation was performed. We discovered that the diketopiperazine derivatives could prevent tubulin assembly through conformational changes. Molecular Mechanics/Poisson–Boltzmann Surface Area (MM-PBSA) calculations showed that the trend of the binding free energies of these inhibitors was in agreement with the trend of their biological activities. Introducing hydrophobic groups into the A-ring was favorable for binding. Energy decomposition indicated that van der Waals interaction played an essential role in the binding affinity of tubulin polymerization inhibitors. In addition, the key residues responsible for inhibitor binding were identified. In summary, this study provided valuable information for development of novel tubulin polymerization inhibitors as anticancer agents.

## Abstract 2

Microtubules are a favorable target for development of anticancer agents.

## Graphical abstract

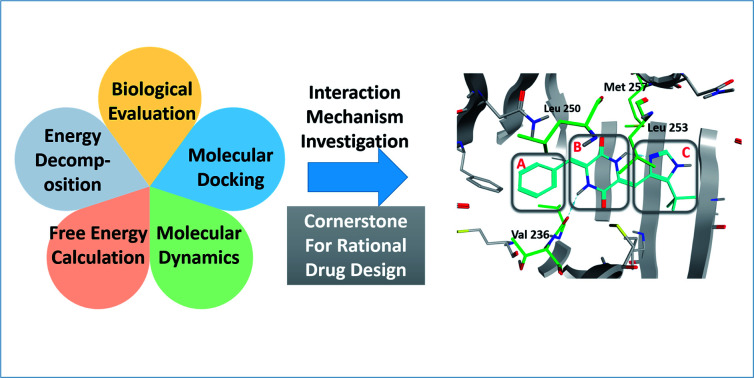

## 1 Introduction

**Fig. 1:**
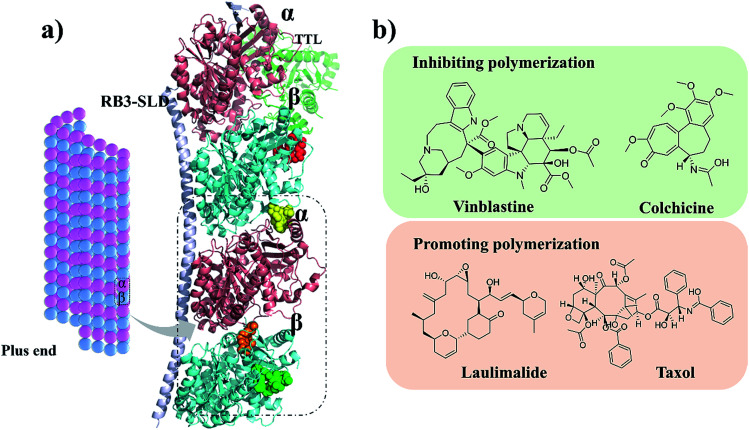
(a) Four microtubule binding sites: colchicine (orange), laulimalide (red), taxol (green) and vinblastine (yellow). α and β tubulins are shown in pink and blue, respectively. (b) Structures of the representative compounds.

Microtubules (MTs) are typically formed by 13 protofilaments associating laterally in parallel to form a hollow and polar cylinder. Each protofilament consists of α,β-tubulin heterodimers assembled with a head-to-tail dynamic configuration.^1–3^ Polymerization dynamics are closely linked to the complex at MT plus end, which switches between phases of growth and disassembly. This so-called ‘‘dynamic instability’’ is controlled by the hydrolysis of GTP in β-tubulin upon polymerization.^4^ As a key component of cytoskeleton, MTs play diverse roles in cell division, cell migration and intracellular transport.^5^ They are considered as potential antitumor targets because destabilizing and stabilizing of MTs can perturb cell division. Currently, as shown in Fig. 1, four binding sites of microtubules have been identified: taxol,^6^ vinblastine,^7^ colchicine^8^ and laulimalide.^9^ Till date, the U.S. Food and Drug Administration (FDA) has approved several anti-tubulin agents targeting the taxol and vinblastine sites in cancer chemotherapy.^10,11^ However, the use of colchicine in cancer chemotherapy is restrained by its low therapeutic index.^12,13^ To overcome the clinical limitations, multiple efforts have been explored to develop the colchicine binding site inhibitors (CBSIs). However, these developed drug candidates failed due to undesirable toxicity and multidrug resistance (MDR). Therefore, it is essential to develop novel CBSIs with low toxicity and high efficacy.

Plinabulin was developed from the natural cyclic diketopiperazine (DKP) derivative ‘‘phenylahistin”, which was isolated from *Aspergillus ustus*.^14,15^ Currently, plinabulin is in phase III clinical trial for treatment of non-small cell lung cancer.^16^ For structure–activity relationship study, a series of diketopiperazine (DKP) derivatives were synthesized and their biological activities were evaluated.^17,18^ Notably, there was a correlation between the dissociation constants of the inhibitors binding to tubulin (*K*_d_) and the IC_50_ values against human colon cancer cell lines HT-29.^17,18^

**Fig. 2:**
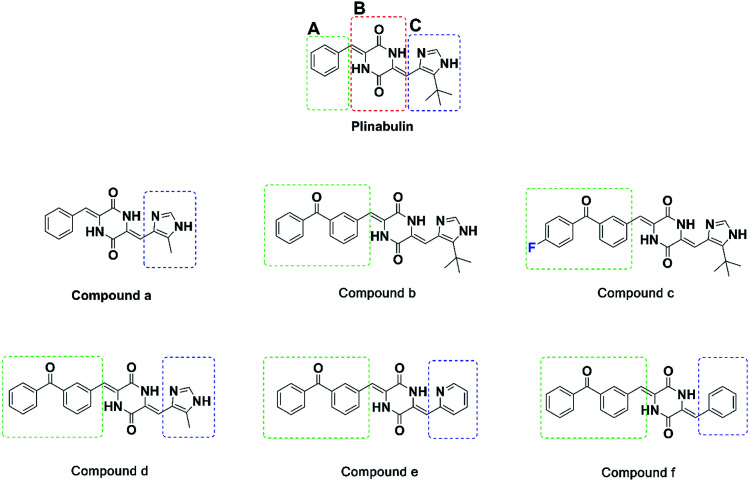
Structures of the synthesized diketopiperazine derivatives.

For further rational drug design, seven DKP derivatives including plinabulin were synthesized (Fig. 2), and their anti-proliferative activities were evaluated against human pancreatic cancer cell line BxPC-3 and human lung cancer cell line NCI-H460. The structure–activity relationship and interaction mechanisms of the derivatives were explored by molecular docking combined with MD simulation. Both structural analysis and binding free energy calculations provided us valuable information for development of novel tubulin polymerization inhibitors as anticancer agents.

## 2 Experimental

### 2.1 Synthesis of the diketopiperazine derivatives

These derivatives were synthesized according to our previous published reaction routes with necessary modification.^19^ The structures were characterized by MS and ^1^H-NMR (see ESI†). The purities of these compounds were determined by HPLC.

### 2.2 SRB assay

Human cancer cell lines NCI-H460 and BxPC-3 were purchased from American Type Cell Culture Collection (ATCC, USA). Sulforhodamine B (SRB) assay was widely used for *in vitro* measurement. Both human lung cancer cell line NCI-H460 and human pancreatic cancer cell line BxPC-3 were cultured in RPMI-1640 media with 2 mM glutamine and 100 U per mL penicillin. Next, BxPC-3 and NCI-H460 cells at logarithmic growth phase were seeded in 96-well plates at 5000 cells per well. Plinabulin derivatives at different concentrations were added into the wells. After incubation for 72 h, 50 μL of stock solution of 50% TCA was added to each well at 4 °C. Then, the 96-well plate was placed at 4 °C for 1 h. Then, 70 μL of 0.4% SRB (w/v) solution in 1% acetic acid was added to each well. The plate was placed at room temperature for 30 min, and then washed with 1% acetic acid five times to remove unbound SRB. Then, 150 μL of 10 mM Trizma base solution was added into each well to solubilize the bound SRB. Finally, the absorbance of 96-well plate was measured using a microplate reader (SpectraMax I3, Molecular Devices, USA) at 540 nm.

### 2.3 *In vitro* tubulin polymerization assay

Tubulin polymerization assay was performed according to the method described by Bonne *et al.* with appropriate modification.^20,21^ A commercial kit (BK011P) was purchased from Cytoskeleton (Danvers, MA, USA). The final tubulin solution contained 80.0 mM piperazine-*N*,*N*′-bis (2-ethanesulfonic acid) sequisodium salt (pH 6.9), 2.0 mM MgCl_2_, 0.5 mM EGTA, 1 mM GTP, and 15% glycerol. The 96-well plate was warmed at 37 °C for 1 min after the compounds (5 μL) were added. Plinabulin was used as a positive control. Then, 45 μL of the tubulin solution was added into each well rapidly, and the plate was read immediately. Fluorescence was monitored (excitation at 360 nm and emission at 450 nm) every 1 min for 20 min by a microplate reader (SpectraMax I3, Molecular Devices, USA).

### 2.4 Immunofluorescence assay

BxPC-3 cells were seeded onto poly-lysine treated coverslips in complete media and treated with 5 nM compound for 24 h. Then, the coverslips were washed three times with PBS. The fixed cells were incubated with 4% paraformaldehyde in PBS at room temperature for 15 min. After rinsing three times with PBS in 5 min and blocking samples in 1% bovine serum albumin for 30 min, the cells were incubated with primary antibody (β-tubulin [1 : 100], Servicebio, Wuhan, China) for 1 h. The nuclei were stained with DAPI (Servicebio, Wuhan, China) as a nuclear counterstain. The cellular microtubule networks were analysed by a confocal microscope. Three random fields per treatment were imaged under a Nikon Eclipse C1 microscope equipped with a Nikon DS-U3. Integrated optical density (IOD) values were recorded, and the images were analysed using Image-Pro Plus 6.0 image analysis software (Media Cybernetics, Inc., Rockville, MD, USA).^22^

### 2.5 Docking method

The X-ray crystal structures of tubulin bound with plinabulin (PDB ID: 5C8Y)^23^ and an in-house prepared co-crystal complex of tubulin with compound b (PDB ID: 5YL4) were used as the starting structures. The three-dimensional structures of the small molecules were generated in Molecular Operating Environment System (MOE) 2016.10 (Chemical Computing Group, Montreal, Canada).^24^ A subsequent energy minimization was carried out using Amber10:EHT force field and R-field solvation. The protein structures were prepared using QuickPrep module of MOE, and the energies were minimized through General method at 0.1 kcal mol^−1^ Å^−2^ RMS Gradient. Initial placement poses were performed with Alpha Triangle placement method. The docking poses were scored using London Δ*G* scoring function with five parameters, such as rotational and translational entropy, ligand flexibility, hydrogen bonding, metal ligations, and desolvation energy. At least 20 poses for each compound were retained and ranked *via* GBVI/WSA Δ*G* scoring function. The reasonable binding model of each compound was selected for further molecular dynamics simulation study.

### 2.6 Molecular dynamics simulation

Molecular dynamics (MD) simulation was performed using GROMACS (version 5.1.4) package and gromos 54a7 force field.^25,26^ Topologies of the proteins were prepared by the pdb2gmx module of GROMACS. The ligand topology files were generated using Automated Topology Builder.^27^ All heterodimer systems were defined in a cubic box at least 1.0 nm from edge, and solvated with SPC water model.^28^ To neutralize the system, NaCl in concentration of 0.1 M was used to replace solvent. Energy of the model proteins was minimized using the steepest descent algorithm and was converged to *F*_max_ (maximum force) < 10.0 kJ mol^−1^. Then, a 100 ps NVT equilibration was carried out under a leap-frog integrator with a 1.4 nm cut-off for van der Waal (vdW) interaction, electrostatic and Linear Constraint Solver (LINCS).^29^ The temperature and pressure systems were regulated by Berendsen temperature^30^ and pressure coupling methods.^31^ After the system was equilibrated by a 100 ps NPT at 300 K, MD simulation with 20 ns was performed with trajectories generated in time step of 2 fs, and the frames were saved every two ps. The convergence of simulation was analysed with several methods, such as root mean square deviation (RMSD), root mean square fluctuation (RMSF), radius of gyration, and number of hydrogen bonds. All coordinate frames from trajectories were superimposed on the initial conformation to remove overall translational and rotational effects. Finally, the MM-PBSA binding free energy was calculated. The images were created with Pymol 0.99.^32^

### 2.7 Free energy calculation

The free energy calculation was performed for 250 snapshots extracted from last 2 ns stable MD trajectory using g_mmpbsa,^33^ which contained all required subroutines from GROMACS and APBS packages. The binding free energy of the protein with ligand in solvent was expressed as follows:Δ*G*_bind_ = *G*_complex_ − (*G*_protein_ + *G*_ligand_)

*G*_complex_ was total free energy of the protein–ligand complex. *G*_protein_ and *G*_ligand_ were total energy of the separated protein and ligand in solvent, respectively. Free energy of each individual *G*_complex_, *G*_protein_ and *G*_ligand_, was estimated as follows:*G*_complex_ = <*E*_MM_> + <*G*_solvation_>

*E*_MM_ is the representation of the average molecular mechanics potential energy in vacuum, and *G*_solvation_ is free energy of solvation. Molecular mechanics potential energy was calculated in vacuum using the following equation:*E*_MM_ = *E*_bonded_ + *E*_nonbonded_ = *E*_bonded_ + (*E*_vdW_ + *E*_elec_)

*E*_bonded_ represents bonding interactions consisting of bond, angle, dihedral and improper interactions. *E*_nonbonded_ interactions involved electrostatic (*E*_elec_) and van der Waals (*E*_vdW_) interactions, which were modelled using Coulomb and Lennard-Jones (LJ) potential functions, respectively. Free energy (*G*_solvation_) of solvation was estimated as the sum of electrostatic solvation free energy (*G*_polar_) and apolar solvation free energy (*G*_nonpolar_):*G*_solvation_ = *G*_polar_ + *G*_nonpolar_

*G*_polar_ and *G*_nonpolar_ are the electrostatic and nonelectrostatic contributions to the solvation free energy, respectively. *G*_polar_ was estimated by solving Poisson–Boltzmann (PB) equation and solvent accessible surface area (SASA). The final Δ*G*_bind_ value was the average value from last 2 ns of the MD simulation.

## 3 Results and discussion

### 3.1 Inhibitory activity assay *in vitro*

The anti-proliferative activities of these compounds were evaluated against BxPC-3 and NCI-H460 cell lines by SRB assay. The values of half maximal inhibitory concentration (IC_50_) were calculated and are summarized in Table 1.

**Table 1:** *In vitro* anti-proliferative activity of DKP derivatives in BxPC-3 and NCI-H460 cell lines, and their inhibition rate of tubulin polymerization at 5 μM^a^

	IC_50_ BxPC-3 (nM)	IC_50_ NCI-460 (nM)	Inhibition rate of tubulin polymerization (5 μM)
Plinabulin	4.4 ± 1.10	26.2 ± 3.20	68.9%
a	306.4 ± 41.40	>1000	12.8%
b	0.9 ± 0.04	4.1 ± 0.60	72.2%
c	0.7 ± 0.03	3.8 ± 1.20	92.5%
d	56.8 ± 4.80	51.7 ± 6.60	66.1%
e	5.0 ± 0.90	27.2 ± 3.10	61.1%
f	91.7 ± 6.00	388.7 ± 53.80	9.9%

a All these values were indicated as the mean ± SD of three independent experiments.

The IC_50_ values of plinabulin were 4.4 nM and 26.2 nM against BxPC-3 cells and NCI-H460 cells, respectively. In comparison with plinabulin, compound b had better anti-proliferative activities; its IC_50_ values were 0.9 nM against BxPC-3 cell lines and 4.1 nM against NCI-H460 cell lines. These results indicated that the benzoyl group substituted on A-ring could enhance the bioactivity of compound b. As shown in Table 1, compound c, with a *para*-F atom at the phenyl group, displayed the highest biological activity, and its IC_50_ values were 0.7 nM and 3.8 nM against BxPC-3 and NCI-H460, respectively.

In contrast, plinabulin had better anti-proliferative activity than that of compound a (IC_50_ = 306 nM for BxPC-3 cells and IC_50_ > 1000 nM for NCI-H460 cells). Similarly, compound b had better anti-proliferative activity than that of compound d (IC_50_ = 56.8 nM for BxPC-3 cell lines, IC_50_ = 51.7 nM for NCI-H460 cells). These results showed that the replacement of *tert*-butyl group with methyl group at C-ring could decrease the bioactivity. Since the substituted group at C-ring could also influence the bioactivity, we further explored compound e with a pyridine group at C-ring, which had comparable bioactivity with compound b (IC_50_ = 5.0 nM for BxPC-3, IC_50_ = 27.2 nM for NCI-H460). However, in contrast, compound f (IC_50_ = 91.7 nM for BxPC-3, IC_50_ = 388.7 nM for NCI-H460) had a benzyl-substituted group at C-ring, and showed greatly reduced bioactivity compared to compound b. These results revealed that the intramolecular hydrogen bond was essential to maintain bioactivity.

**Fig. 3:**
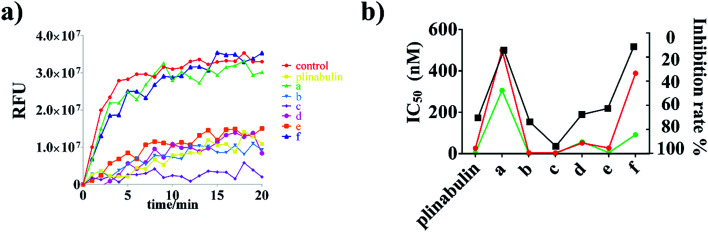
Effects of plinabulin and compounds a–f at 5 μM on the tubulin polymerization. (a) Tubulin polymerization inhibition activities measured with time at 37 °C. (b) Comparison of IC_50_ values (BxPC-3: green; NCI-H460: red) and tubulin inhibition rate (black) of DKP derivatives.

To demonstrate the binding activity, fluorescence-based tubulin polymerization assay was measured with time (Fig. 3a). Inhibition rates of plinabulin and compounds a–f were found to be 68.9%, 12.8%, 72.2%, 92.5%, 66.1%, 61.1% and 9.9%, respectively (Table 1). In contrast, the polymerization inhibition activities were consistent with the IC_50_ values against BxPC-3 and NCI-H460 cell lines as shown in Fig. 3b.

**Fig. 4:**
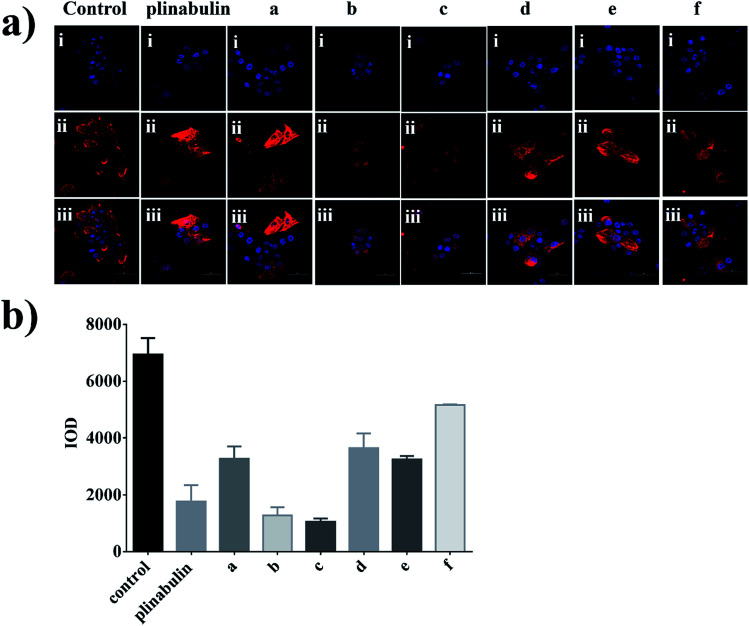
Immunofluorescence assay of plinabulin and compounds a–f inhibition of tubulin polymerization *in vitro*. (a) Confocal images of DKP derivatives (5 nM) disrupting the mitotic spindles in BxPC-3 cell. (i) Nuclear (blue); (ii) tubulin (red); (iii) (i) and (ii) were overlapped. Scale bar is 50 μm. (b) Semi-quantitative analysis of the inhibition of tubulin polymerization.

An immunofluorescence assay was performed to confirm whether compounds a–f could disrupt the microtubule dynamics in cells. As shown in Fig. 4a, the microtubule network in BxPC-3 cells was well-defined and wrapped around the uncondensed cell nucleus; in contrast, the formation of spindles in cells after exposure to the compounds demonstrated distinct abnormalities. Furthermore, semi-quantitative analyses of these compounds exhibited the disruption of tubulin polymerizations as shown in Fig. 4b, which could be considered as direct evidence. Again, the inhibition activities were also consistent with the anti-proliferative activities as shown in Fig. 3b.

### 3.2 Docking results

**Fig. 5:**
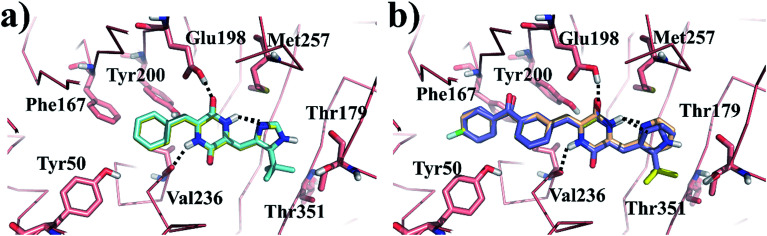
The reasonable binding poses of the inhibitors in tubulin. (a) Plinabulin and compound a in the pocket 5C8Y. (b) Compounds b–f in the pocket 5YL4.

Molecular docking simulation of plinabulin and compounds a–f were performed using the dock module of MOE software package. The reasonable binding poses in colchicine binding pocket are displayed in Fig. 5. The docking results indicated that all derivatives formed a crucial hydrogen bonding interaction between the diketopiperazine backbone and Val236 of β tubulin. For MD simulation, considering the flexibility of tubulin, the pocket of 5C8Y was selected for the binding poses of plinabulin and compound a, while the pocket of 5YL4 was selected for the binding poses of compounds b–f.

### 3.3 Structural stability

**Fig. 6:**
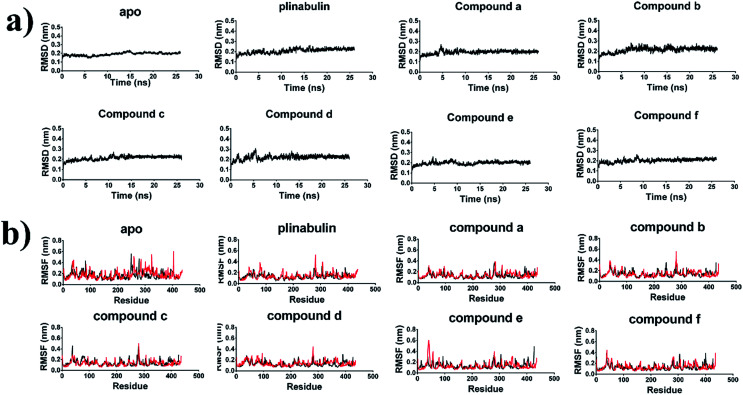
Structural stability of the tubulin-apo and tubulin-inhibitors complexes. (a) Backbone RMSD of dipolymer (black). (b) RMSF of side chain in α-tubulin (red) and β-tubulin (black).

It was crucial to obtain a stable molecular dynamics trajectory for subsequent analysis. The time dependent root mean square deviation (RMSD) of the backbone was calculated to evaluate the structural stability. As shown in Fig. 6a, all systems reached stability after 22 ns. The residues-dependent root mean squared fluctuation (RMSF) of the side chain was calculated to assess the flexibility of residues. In general, the RMSF plots presented low fluctuations in α-helix and β-sheet, and high peaks in the loops. Moreover, H1–B2 loop (residues 35–60) and M-loop (residues 272–290) were involved in contacting the protofilaments^34^ and had higher structural flexibility (Fig. 6b).

### 3.4 Structural analysis

**Fig. 7:**
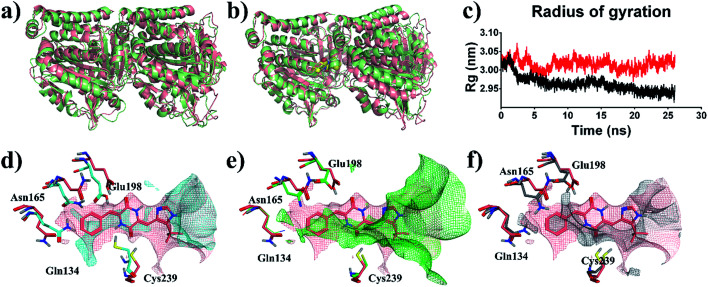
Structural analysis of MD simulation. Overview of the structures of (a) tubulin-apo at 0 ns (pink) and 26 ns (light green); (b) tubulin–plinabulin at 0 ns (pink) and 26 ns (light green); (c) radius of gyration of tubulin–plinabulin (red) and tubulin-apo (black) with time; (d) the pocket comparison of tubulin-apo of 0 ns (pink) and 26 ns (cyan); (e) the pocket comparison of the crystal structures of tubulin bound with plinabulin (pink) (PDB ID: 5C8Y) and with colchicine (green) (PDB ID: 1SA0); (f) the pocket comparison of 5C8Y (pink) and tubulin-apo (grey) (PDB ID: 3HKB).

To reveal the effects of DKP derivatives on the overall structure of tubulin, the structures of tubulin-apo and tubulin–plinabulin were superimposed before and after molecular dynamics simulation (Fig. 7a or b). In comparison with tubulin-apo, the conformation of tubulin–plinabulin clearly changed. Furthermore, the gyration radii of tubulin-apo and tubulin–plinabulin were 2.93 and 3.02 nm, respectively (Fig. 7c). The possible reason was that the DKP derivatives could inhibit the microtubules' polymerization through altering the conformation of tubulin. As shown in Fig. 7d, the pink surface and the cyan surface represent the plinabulin binding pocket before and after MD simulation, respectively. The binding pocket of plinabulin in tubulin-apo faded away after 26 ns of MD simulation. In addition, the crystal structures of tubulin with colchicine (PDB ID: 1SA0 (ref. 8)) and plinabulin (PDB ID: 5C8Y^23^) were superposed with β-tubulin (Fig. 7e). This indicated that plinabulin was slightly overlapped with colchicine in the binding site and the active site pockets showed that plinabulin could not effectively fit into the colchicine pocket. Therefore, we assumed that the binding pocket of plinabulin was induced and did not exist in original tubulin polymers. In order to verify this, the structures of tubulin without any ligand (PDB ID: 3HKB^35^) and tubulin–plinabulin were superimposed with β-tubulin. As shown in Fig. 7f, no binding pockets were observed for plinabulin in 3HKB or in the simulated tubulin-apo structure after 26 ns, which conformed to our assumption.

**Fig. 8:**
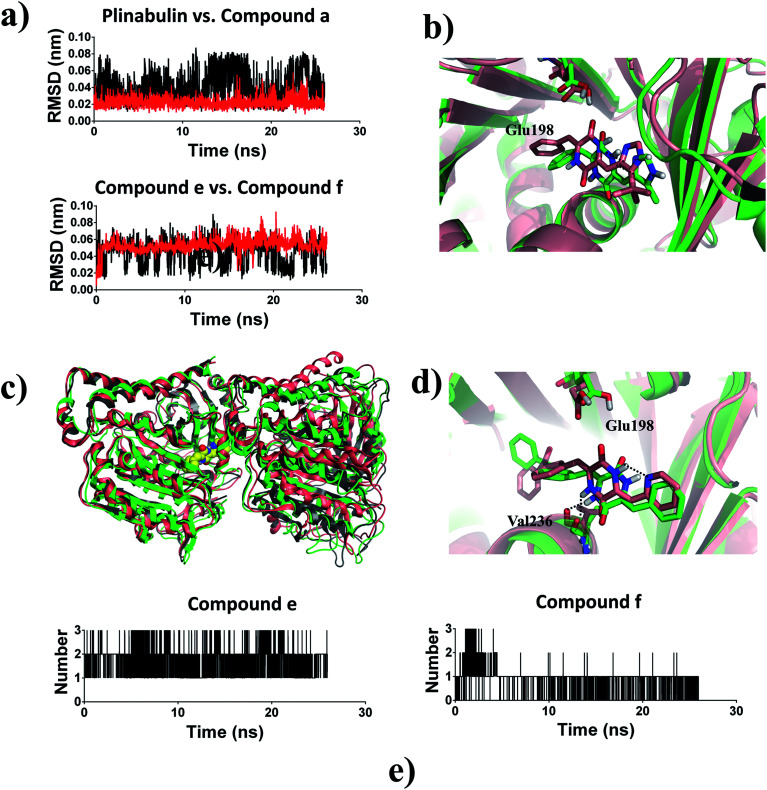
(a) Ligand-position RMSDs of plinabulin, compound a, compound e, and compound f; (b) structure analysis of MD simulation: tubulin–plinabulin (pink) *vs.* tubulin–a (green) (c) overview of the structures of tubulin–e (pink) and tubulin–f (green) after MD simulation *vs.* tubulin–f (grey) before MD simulation; (d) structure analysis of MD simulation: tubulin–e (pink) *vs.* tubulin–f (green); (e) number of hydrogen bonds: compound e*vs.* compound f.

Based on the ligand-position RMSD results, plinabulin with *t*-butyl moiety was more stable than compound a with methyl moiety during MD simulation (Fig. 8a). A migration of compound a was observed when the structure of tubulin–a complex was superimposed onto the structure of tubulin–plinabulin complex (Fig. 8b). The structural analysis indicated that compound a shifted outward and the methyl group shifted toward the corresponding *t*-butyl group location. We assumed that the *t*-butyl moiety could interact with tubulin in a hydrophobic manner, which was the major contribution to the inhibitor's binding.

Compound e with an intramolecular hydrogen bond was stable in the pocket compared with compound f, which lacked the intramolecular hydrogen bond (Fig. 8a). The structure of tubulin–f complex had a deflection in B-ring and C-ring compared with the tubulin–e complex (Fig. 8d). This deflection made compound f lose the hydrogen bond with Glu198; this result was also proved by analysis of the number of hydrogen bonds (Fig. 8e). The overall structural superimposition indicated that the tubulin–f complex was stable before and after MD simulation, while the tubulin–e complex was bent greatly after MD simulation (Fig. 8c), a conformation change which could hinder the assembly of tubulin.^36^ These results were consistent with the biological assay that compound e had better anti-proliferative and inhibition activities of microtubules in comparison with compound f. Therefore, we assumed that the intramolecular hydrogen bond of this ligand was important for forming a reasonable binding mode and further inhibiting tubulin polymerization.

### 3.5 Binding free energy calculation

Combined with MD simulation, the binding free-energy calculation method has become a powerful tool for quantitative determination of the protein–ligand interaction. In total, 250 snapshots extracted from last 2 ns of MD trajectories were used to analyse the binding free energy with MM-PBSA method. As shown in Table 2, the binding free energy (Δ*G*_bind_) of plinabulin and compounds a–f were −100.82, −83.96, −171.32, −193.86, −92.47, −148.09, and −146.15 kJ mol^−1^, respectively.

**Table 2:** The energetic terms obtained from MM-PBSA calculation for DKP derivatives binding to tubulin (kJ mol^−1^)

	Δ*E*_vdW_	Δ*E*_ele_	Δ*G*_PB_	Δ*G*_SA_	Δ*G*_bind_
Plinabulin	−173.09 ± 1.60	−14.22 ± 2.85	106.19 ± 2.26	−19.81 ± 0.19	−100.82 ± 2.05
Compound a	−174.66 ± 1.99	−18.29 ± 1.26	128.65 ± 2.72	−19.60 ± 0.24	−83.96 ± 3.33
Compound b	−256.99 ± 2.04	−26.93 ± 1.12	139.42 ± 1.39	−26.95 ± 0.29	−171.32 ± 2.91
Compound c	−281.62 ± 2.80	−18.79 ± 1.49	131.62 ± 2.20	−25.07 ± 0.19	−193.86 ± 3.82
Compound d	−222.69 ± 2.50	−14.61 ± 1.92	169.84 ± 2.54	−25.09 ± 0.24	−92.47 ± 2.45
Compound e	−239.83 ± 3.37	−30.23 ± 2.48	146.88 ± 2.37	−24.74 ± 0.27	−148.09 ± 3.45
Compound f	−231.46 ± 1.84	−36.86 ± 2.60	146.78 ± 3.03	−24.64 ± 0.24	−146.15 ± 3.34

According to energy components of the binding free energy, the major favourable contributor of the inhibitor binding was van der Waals interaction (Δ*E*_vdW_). Moreover, the electrostatic energy (Δ*E*_ele_) and nonpolar solvation free energy (Δ*G*_SA_) were also favourable for the binding. In addition, the non-polar interactions (Δ*E*_vdW_ + Δ*G*_SA_) were the dominating force for inhibitor binding. These results suggested that optimization of van der Waals interactions between inhibitors and tubulin might improve their biological activities. Therefore, we concluded that hydrophobic interaction played an important role in the development of potent tubulin polymerization inhibitors.

**Fig. 9:**
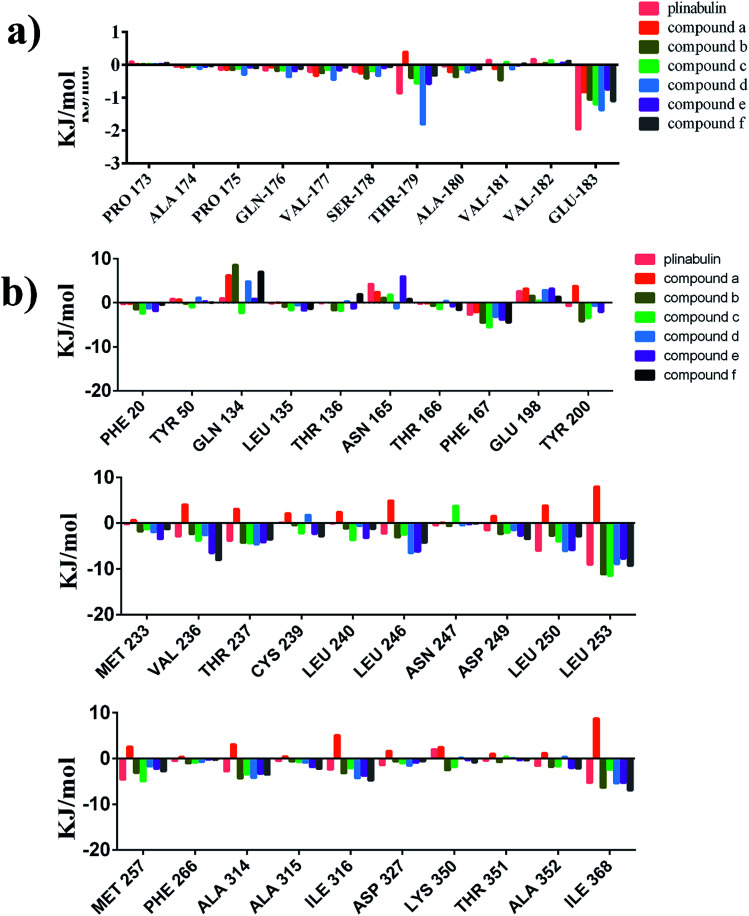
Contributions of the key residues of plinabulin and compounds a–f. (a) α-tubulin. (b) β-tubulin.

To identify the key residues for these inhibitors binding to tubulin, the binding free energies were decomposed into individual residues. Δ*G*_bind_ energy contributions from each residue are represented in Fig. S1.† The key residues are summarized in Fig. 9. The results revealed that the attractive contributions primarily originate from Glu183 of α-tubulin and β-tubulin residues, such as Asn165, Tyr200, Asp249, Leu253, Met257, Ala314, Ile316, and Ile368. Docking results indicated that DKP ring could form a hydrogen bond with the protonated carboxyl group of Glu198 of β-tubulin. However, the energy decomposition indicated that DKP ring had unfavourable interaction with Glu198 of β-tubulin, which probably resulted from the electron repulsion between the other carboxylic oxygen of Glu198 and DKP ring (Fig. S2†). The potency could be improved by removing this unfavourable interaction.

## 4 Conclusion

In this study, both molecular docking and MD simulation were used to explore the interaction between tubulin and DKP derivatives. The binding free energy decomposition showed that van der Waals interactions were dominant for the binding affinity. Further, the total binding free energy was decomposed into each residue. The binding free energy calculation revealed that the binding affinity between ligand and tubulin was mostly contributed from β-tubulin. The results also indicated that the key residues for tubulin binding were Asn165, Tyr200, Val236, Asp249, Leu253, Met257, Ala314, Ile316, and Ile368, which created a hydrophobic pocket at β-tubulin. In addition, the trend of the binding free energy of these inhibitors was in agreement with the trend of their biological activities (Table 1).

In summary, this investigation provided a molecular level understanding of the mechanism, and provided valuable information for further rational anticancer drug design.

## Conflicts of interest

There are no conflicts to declare.

## Abbreviations

MD: Molecular Dynamics
MM-PBSA: Molecular Mechanics/Poisson–Boltzmann Surface Area
vdW: van der Waal
CBSI: Colchicine Binding Site Inhibitor
MTs: Microtubules
MTAs: Microtubule Targeting Agents
FDA: Food and Drug Administration
MDR: Multidrug Resistance
IOD: Integrated Optic Density
PDB: Protein Data Bank
SRB: Sulforhodamine B
MS: Mass Spectrometry
NMR: Nuclear Magnetic Resonance
HPLC: High Performance Liquid Chromatography

## Supplementary Material

RA-008-C7RA12173C-s001
