# Supplementary material for: Biological activity and interaction mechanism of the diketopiperazine derivatives as tubulin polymerization inhibitors
Source: RSC Adv. 2018 Jan 3;8(2):1055–64. doi: 10.1039/c7ra12173c (PMC9076956; doi:10.1039/c7ra12173c)
Supplement: RA-008-C7RA12173C-s001 [file RA-008-C7RA12173C-s001.pdf]

Electronic Supplementary Material (ESI) for RSC Advances.
This journal is © The Royal Society of Chemistry 2017

Supporting Information

(Total of 15 pages)

for

Biological activity and interaction mechanism of the diketopiperazine derivatives as tubulin polymerization inhibitors

Zhenhua Tian, [a] Yanyan Chu, [a, c] Hui Wang, [a] Lili Zhong, [a] Mengyan Deng, [a] Wenbao Li* [a, b, c]

a) School of Medicine and Pharmacy, Ocean University of China, Qingdao 266003, China

b) Innovation Center for Marine Drug Screening and Evaluation, Qingdao National Laboratory for Marine Science and Technology, Qingdao 266071, China

c) Marine Biomedical Research Institute of Qingdao, Qingdao 266071, China

Table of contents

Synthesis routine and characterization of DKP derivatives (Page 4-13)

Fig. S1. Spectra of the inhibitor–residue interaction for tubulin-DKP derivative complexes by α or β tubulin.

Fig. S2 Binding mode of plinabulin with tubulin.

Scheme:

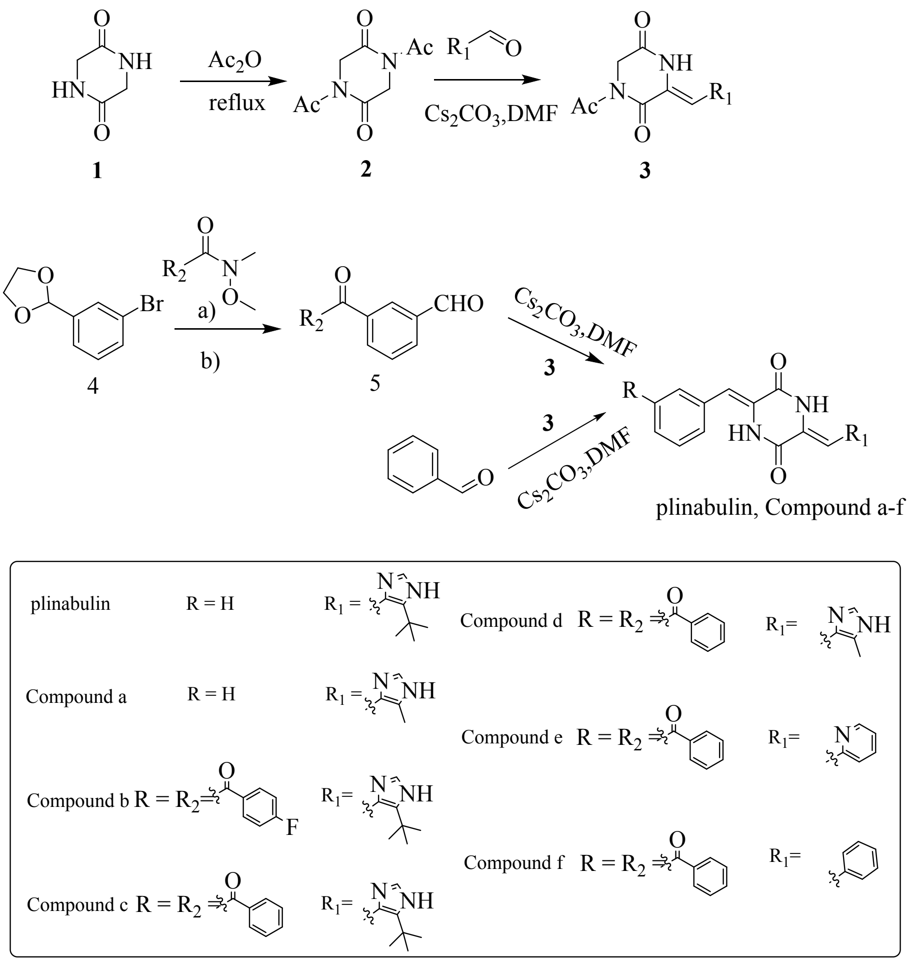

Regents and conditions
(a) n-BuLi, THF, -78 ^{o}C; (b) HCl (1M), THF

1,4-diacetylpiperazine-2,5-dione (2)

The mixture of glycine anhydride (15 g, 131.4 mmol) and acetic anhydride (54 g, 52.5 mmol) was stirred at reflux temperature for 7 h and the reaction was monitored using TLC. After reaction completed, solvent was removed under reduced pressure and the residue obtained was re-crystallized from ethyl acetate/diethyl ether to yield the desired product. (21.6 g, 85%)

(Z)-1-acetyl-3-((5-(tert-butyl)-1H-imidazol-4-yl)methylene)piperazine-2,5-dione (3)

(R_1 = 5-(tert-butyl)-1H-imidazole)

The mixture of compound 2 (5 g, 32.85 mmol), Cs_2CO_3 (16.05 g, 49.28 mmol), aldehyde (13.02 g, 65.7 mmol) and DMF (35 mL) was stirred at room temperature under N_2 atmospher for 24 h and the

reaction was monitored using TLC. After reaction was completed, the mixture was poured into crashed ice and the solid was filtered to give product (Yield: 4.05 g, 42%)

(Z)-1-acetyl-3-((5-(tert-butyl)-1H-imidazol-4-yl)methylene)piperazine-2,5-dione (3)

(R_1 = 5-methyl-1H-imidazole)

The mixture of compound 2 (2.5 g, 16.43 mmol), Cs_2CO_3 (8.0 g, 24.65 mmol), aldehyde (6.51 g, 32.85 mmol) and DMF (18 mL) was stirred at room temperature under N_2 atmospher for 24 h and the reaction was monitored using TLC. After reaction was completed, the mixture was poured into crashed ice and the solid was filtered to give product (Yield: 2 g, 42%)

(Z)-1-acetyl-3-((5-(tert-butyl)-1H-imidazol-4-yl)methylene)piperazine-2,5-dione (3)

(R_1 = pyridine)

The mixture of compound 2 (3.7 g, 18.7 mmol), Cs_2CO_3 (16.05 g, 49.28 mmol), aldehyde (1 g, 9.3 mmol) and DMF (35 mL) was stirred at room temperature under N_2 atmospher for 24 h and the reaction was monitored using TLC. After reaction was completed, the mixture was poured into crashed ice and the solid was filtered to give product (Yield: 560 g, 24%)

(Z)-1-acetyl-3-((5-(tert-butyl)-1H-imidazol-4-yl)methylene)piperazine-2,5-dione (3)

(R_1 = benzene)

The mixture of compound 2 (3.2 g, 21.06 mmol), Cs_2CO_3 (25 g, 10.29 mmol), aldehyde (13.02 g, 8.24 mmol) and DMF (22 mL) was stirred at room temperature under N_2 atmospher for 24 h and the reaction was monitored using TLC. After reaction was completed, the mixture was poured into crashed ice and the solid was filtered to give product (Yield: 2.6 g, 39%)

(Z)-3-((Z)-benzylidene)-6-((5-(tert-butyl)-1H-imidazol-4-yl)methylene)piperazine-2,5-dione (Plinabulin)

The mixture of compound 3 (132 mg, 0.84 mmol), Cs_2CO_3 (220 g, 0.65 mmol), aldehyde (145 g, 0.65 mmol) and DMF (10 mL) was stirred at 50 °C under N_2 atmospher for 24 h and the reaction was monitored using TLC. After reaction was completed. The mixture was extracted with EtOAc. The organic layer was washed with brine and was dried over anhydrous sodium sulfate (Na_2SO_4). Then solvent was removed under reduced pressure. The residue was purified by silica gel column chromatography give product (Yield: 86 mg, 43%) HPLC (UV=254): 99%. LC-MS: $[M+H]^+ = 337$. ^1H NMR (500 MHz, DMSO-d_6) δ 12.33 (s, 1H), 12.25 (s, 1H), 10.05 (s, 1H), 7.85 (s, 1H), 7.53 (d, $J = 7.7$ Hz, 2H), 7.41 (t, $J = 7.6$ Hz, 2H), 7.31 (t, $J = 7.4$ Hz, 1H), 6.85 (s, 1H), 1.38 (s, 10H).

(Z)-3-((Z)-benzylidene)-6-((5-methyl-1H-imidazol-4-yl)methylene)piperazine-2,5-dione (Compound a)

According to the procedure described for the synthesis of plinabulin. (Yield: 28 mg, 43%) HPLC (UV=254): 97%. LC-MS: $[M+H]^+ = 295$. ^1H NMR (500 MHz, DMSO-d_6) δ 11.70 (s, 1H), 11.15 (s, 1H), 9.16 (s, 1H), 7.05 (s, 1H), 6.71 (d, $J = 7.7$ Hz, 2H), 6.60 (t, $J = 7.7$ Hz, 2H), 6.51 (d, $J = 7.4$ Hz, 1H), 5.93 (s, 1H), 5.77 (s, 1H), 1.50 (s, 3H).

(Z)-3-((Z)-3-benzoylbenzylidene)-6-((5-(tert-butyl)-1H-imidazol-4-yl)methylene)piperazine-2,5-dione (Compound b)

To a cooled (-78 °C) solution of 2.5 M n-BuLi (2 mL) in THF (3 mL) was added 1-bromo-3-(1,3-dioxolan-2-yl)-benzene (4) (578 mg, 2.52 mmol) in anhydrous THF (5 mL), and the mixture was stirred for 40 min. Weinreb amides (500 mg, 3.03 mmol) was added dropwise at -78 °C. After the reaction was completed (about 2 hour), the mixture was treated with saturated NH_4Cl (aq) and extracted with EtOAc. The organic layer was washed with brine and was dried over anhydrous sodium sulfate (Na_2SO_4). Then solvent was removed under reduced pressure. The residue was purified by silica gel column chromatography give product (Yield: 400 mg, 62%). Step b: The mixture of compound obtained from step a, (400 mg, 1.57 mmol), 1M HCl (7.8 mL), and THF (10

mL) was stirred at room temperature and the reaction was monitored using TLC. After reaction was completed, The mixture was extracted with EtOAc. The organic layer was washed with brine and was dried over anhydrous sodium sulfate (Na_2SO_4). Then solvent was removed under reduced pressure. The residue was purified by silica gel column chromatography give product. The mixture of compound 3 (123 mg, 0.42 mmol), Cs_2CO_3 (209 mg, 0.64mmol), aldehyde (134 mg, 0.64 mmol) and DMF (5 mL) was stirred at 50 ^oC under N_2 atmospher for 24 h and the reaction was monitored using TLC. After reaction was completed. The mixture was extracted with EtOAc. The organic layer was washed with brine and was dried over anhydrous sodium sulfate (Na_2SO_4). Then solvent was removed under reduced pressure. The residue was purified by silica gel column chromatography give product (Yield: 80 mg, 43%). HPLC (UV=254): 97%. LC-MS: $[M+H]^+$ = 441. ^1H NMR (500 MHz, DMSO-d_6) δ 12.33 (s, 1H), 12.28 (s, 1H), 10.34 (s, 1H), 7.86 – 7.80 (m, 4H), 7.75 (d, J = 7.7 Hz, 1H), 7.69 (t, J = 7.4 Hz, 1H), 7.64 (d, J = 7.7 Hz, 1H), 7.58 (t, J = 7.7 Hz, 3H), 6.86 (s, 1H), 6.80 (s, 1H), 1.38 (s, 8H).

(Z)-3-((5-(tert-butyl)-1H-imidazol-4-yl)methylene)-6-((Z)-3-(4-fluorobenzoyl)benzylidene) piperazine-2,5-dione (Compound c)

According to the procedure described for the synthesis of **Compound b**. (Yield: 46 mg, 48%). HPLC (UV=254): 98%. LC-MS: $[M+H]^+$ = 459. ^1H NMR (500 MHz, DMSO-d_6) δ 12.33 (s, 1H), 12.28 (s, 1H), 10.33 (s, 1H), 7.91 (dd, J = 8.4, 5.7 Hz, 2H), 7.85 (s, 1H), 7.82 (s, 1H), 7.75 (d, J = 7.6 Hz, 1H), 7.63 (d, J = 7.6 Hz, 1H), 7.58 (t, J = 7.6 Hz, 1H), 7.40 (t, J = 8.7 Hz, 2H), 6.86 (s, 1H), 6.80 (s, 1H), 1.38 (s, 9H).

(Z)-3-((Z)-3-benzoylbenzylidene)-6-((5-methyl-1H-imidazol-4-yl)methylene)piperazine-2,5-dione (Compound d)

According to the procedure described for the synthesis of **Compound b**. (Yield: 62 mg, 46%). HPLC (UV=254): 98%. LC-MS: $[M+H]^+$ = 399. ^1H NMR (500 MHz, DMSO-d_6) δ 11.98 (s, 1H),

7.86 (d, J = 8.0 Hz, 2H), 7.81 (s, 2H), 7.78 (d, J = 7.6 Hz, 1H), 7.70 (t, J = 7.4 Hz, 1H), 7.63 (d, J = 7.7 Hz, 1H), 7.61 – 7.56 (m, 3H), 6.78 (s, 1H), 6.58 (s, 1H), 2.32 (s, 3H).

(Z)-3-((Z)-3-benzoylbenzylidene)-6-(pyridin-2-ylmethylene)piperazine-2,5-dione (Compound e)

According to the procedure described for the synthesis of **Compound b**. (Yield: 30 mg, 37%). HPLC (UV=254): 97%. LC-MS: $[M+H]^+$ = 396. ^1H NMR (500 MHz, DMSO-d_6) δ 12.60 (s, 1H), 12.60 (s, 1H), 10.65 (s, 1H), 8.73 (d, J = 4.4 Hz, 1H), 7.91 (td, J = 7.8, 1.6 Hz, 1H), 7.87 (s, 1H), 7.83 (d, J = 7.3 Hz, 2H), 7.79 (d, J = 7.7 Hz, 1H), 7.73 – 7.65 (m, 3H), 7.59 (dd, J = 13.8, 7.5 Hz, 3H), 7.38 (dd, J = 7.0, 5.3 Hz, 1H), 6.90 (s, 1H), 6.73 (s, 1H).

(3-((Z)-3-benzoylbenzylidene)-6-((Z)-benzylidene)piperazine-2,5-dione) (Compound f)

According to the procedure described for the synthesis of Compound b. (Yield: 12 mg, 29%). HPLC (UV=254): 98%. LC-MS: $[M+H]^+$ = 395 ^1H NMR (500 MHz, DMSO-d_6) δ 9.61 (d, J = 82.9 Hz, 2H), 7.04 (s, 1H), 7.01 (d, J = 7.5 Hz, 2H), 6.96 (d, J = 7.3 Hz, 2H), 6.78 (dd, J = 7.6, 4.4 Hz, 3H), 6.73 (d, J = 7.5 Hz, 3H), 6.60 (t, J = 7.6 Hz, 3H), 6.51 (t, J = 7.3 Hz, 1H), 6.02 (s, 1H), 5.96 (s, 1H).

(Z)-3-((Z)-benzylidene)-6-((5-(tert-butyl)-1H-imidazol-4-yl)methylene)piperazine-2,5-dione (Plinabulin)

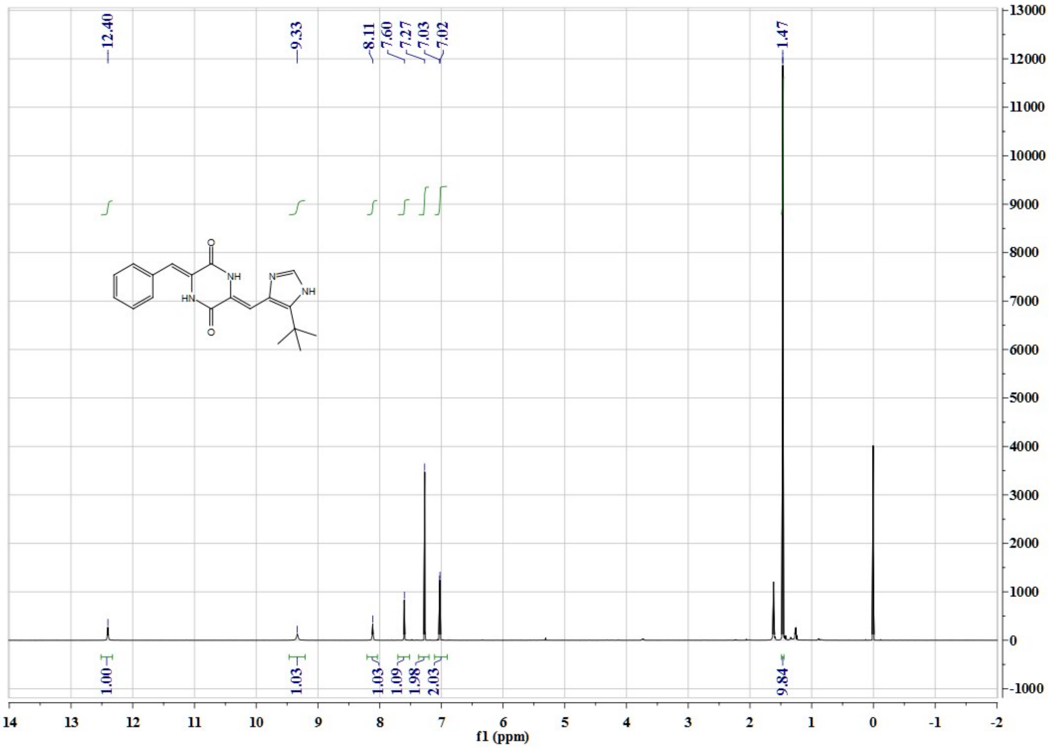

(Z)-3-((Z)-benzylidene)-6-((5-methyl-1H-imidazol-4-yl)methylene)piperazine-2,5-dione

(Compound a)

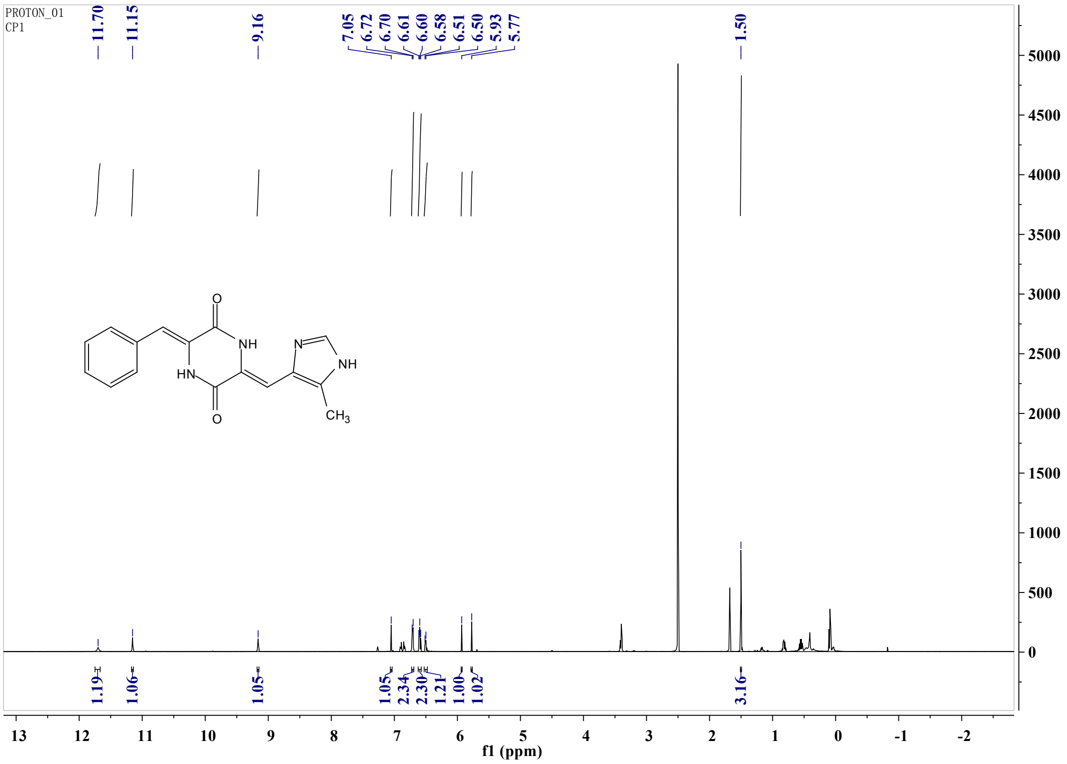

(Z)-3-((Z)-3-benzoylbenzylidene)-6-((5-(tert-butyl)-1H-imidazol-4-yl)methylene)piperazine-2,5-dione (Compound b)

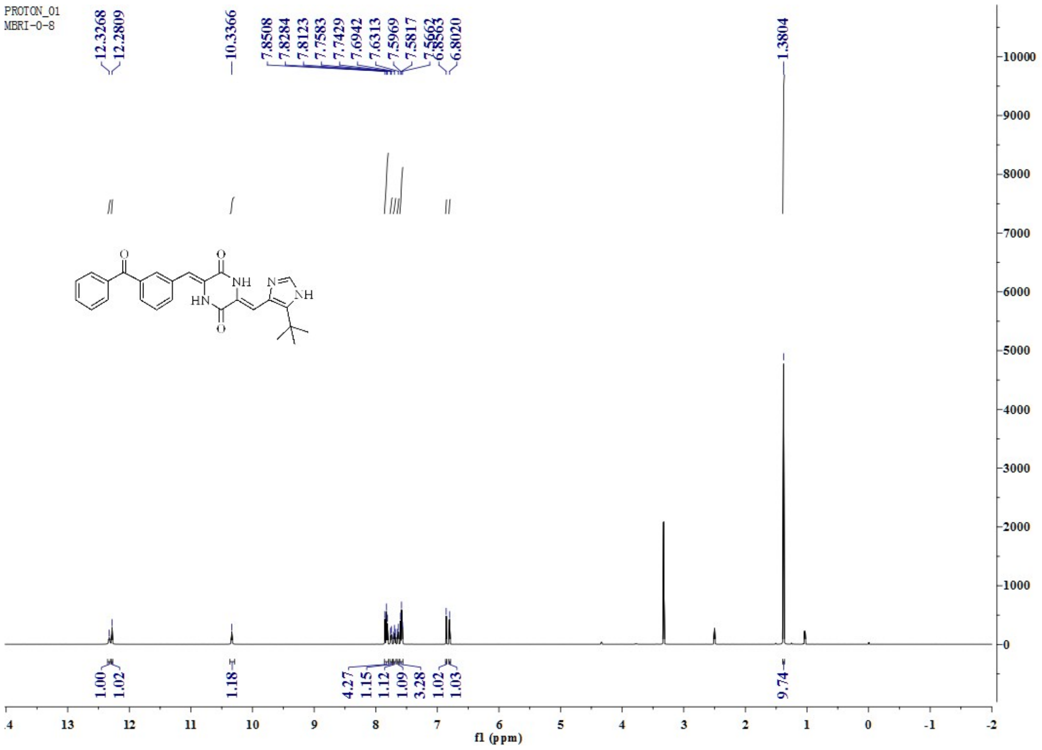

(Z)-3-((5-(tert-butyl)-1H-imidazol-4-yl)methylene)-6-((Z)-3-(4-fluorobenzoyl)benzylidene) piperazine-2,5-dione (Compound c)

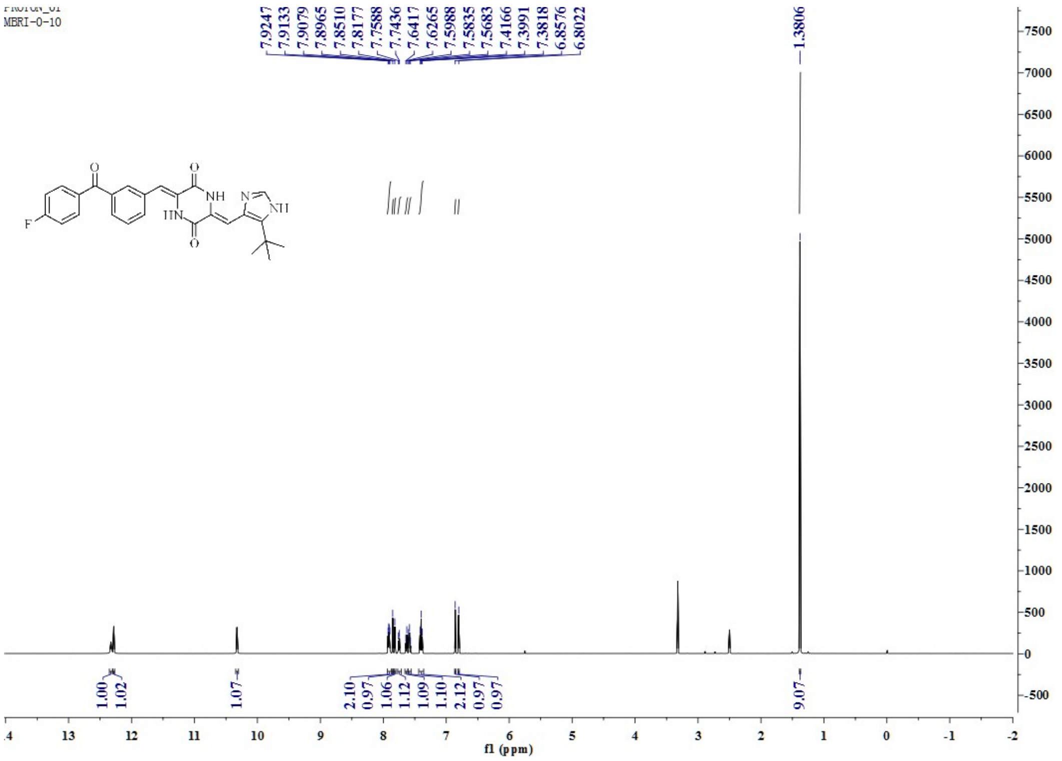

(Z)-3-((Z)-3-benzoylbenzylidene)-6-((5-methyl-1H-imidazol-4-yl)methylene)piperazine-2,5-dione (Compound d)

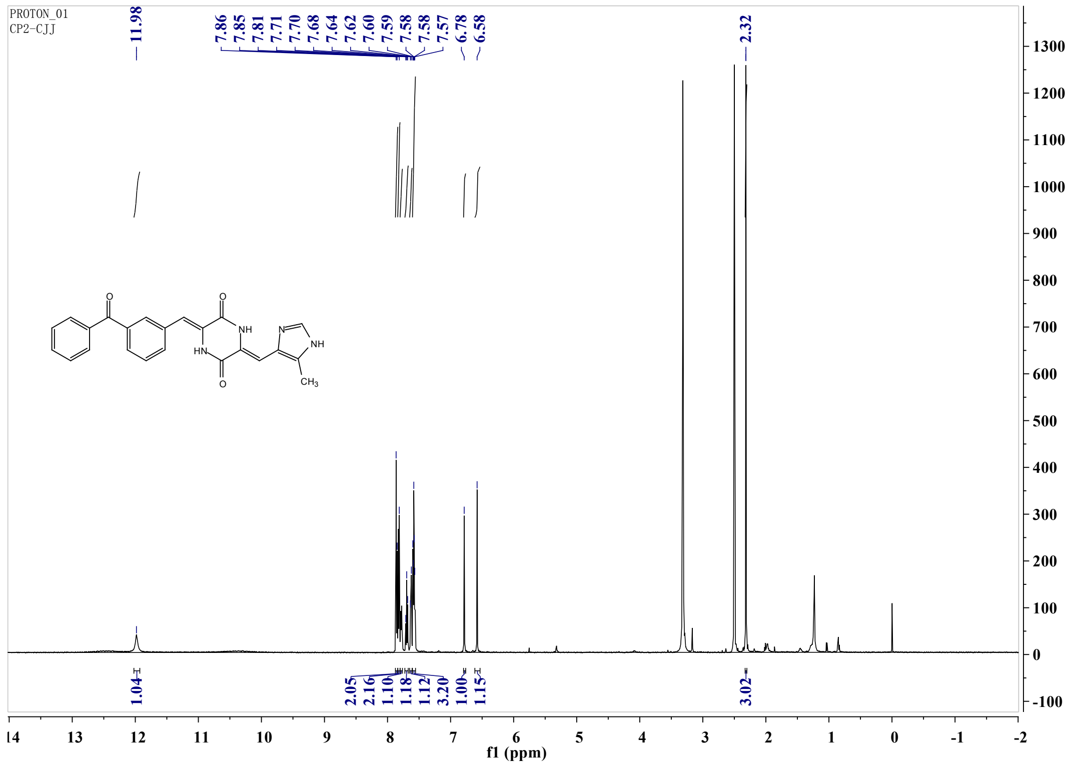

(Z)-3-((Z)-3-benzoylbenzylidene)-6-(pyridin-2-ylmethylene)piperazine-2,5-dione (Compound e)

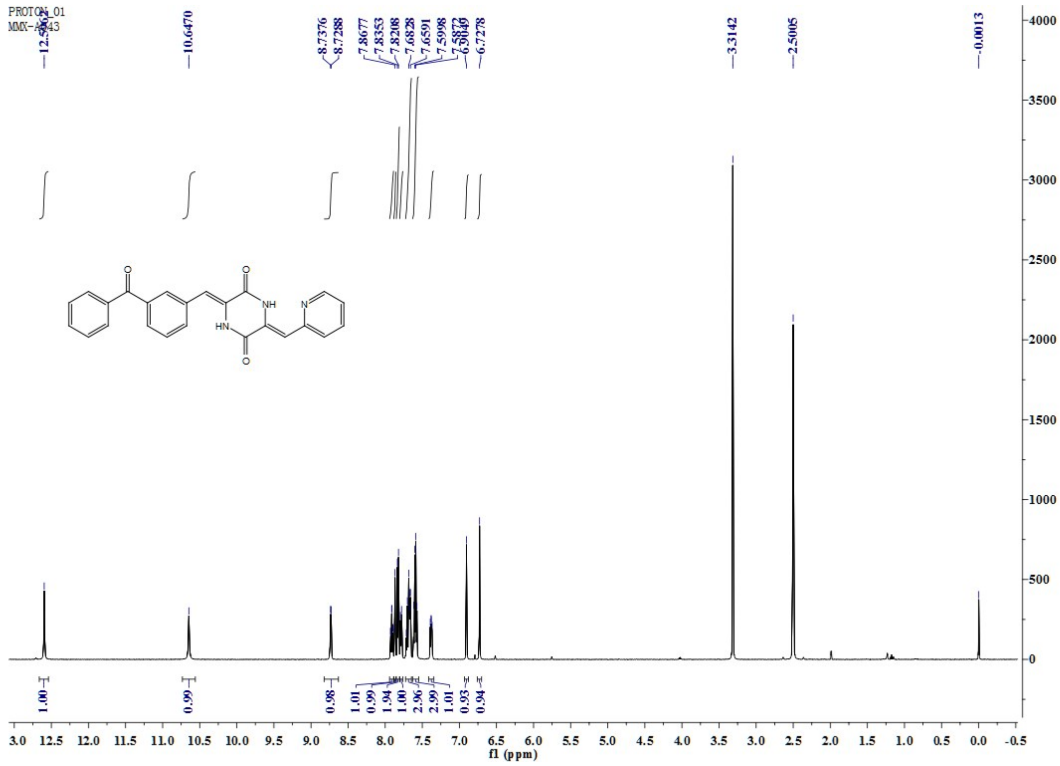

(3-((Z)-3-benzoylbenzylidene)-6-((Z)-benzylidene)piperazine-2,5-dione) (Compound f)

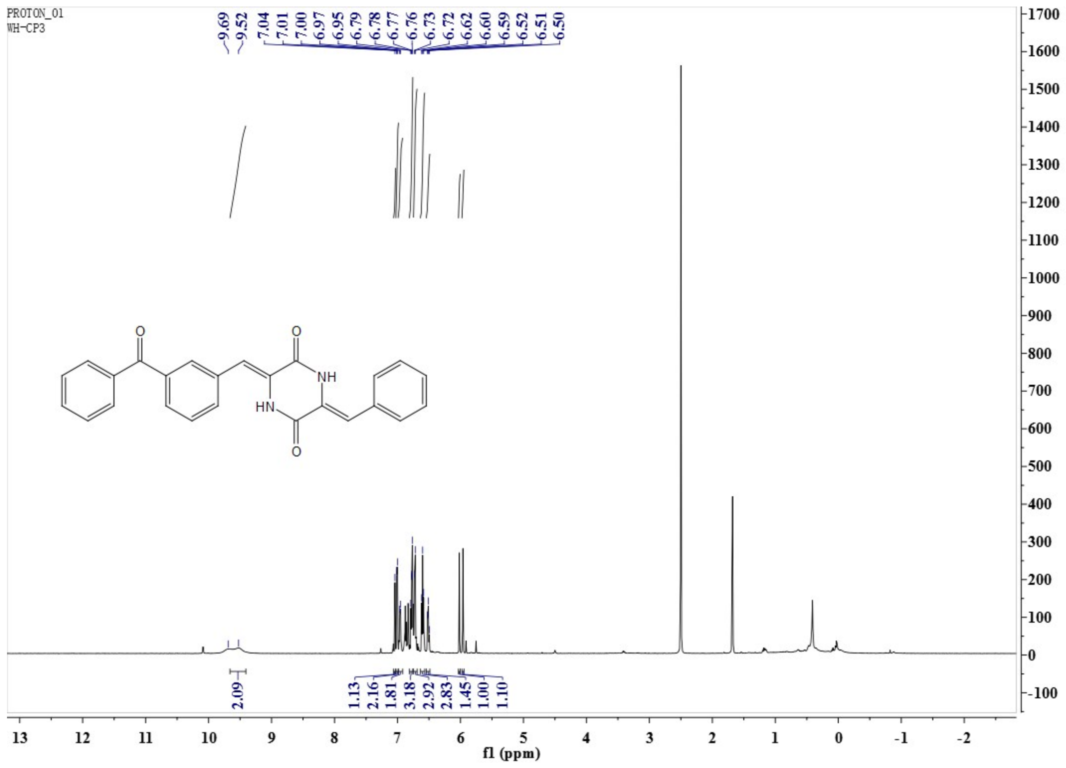

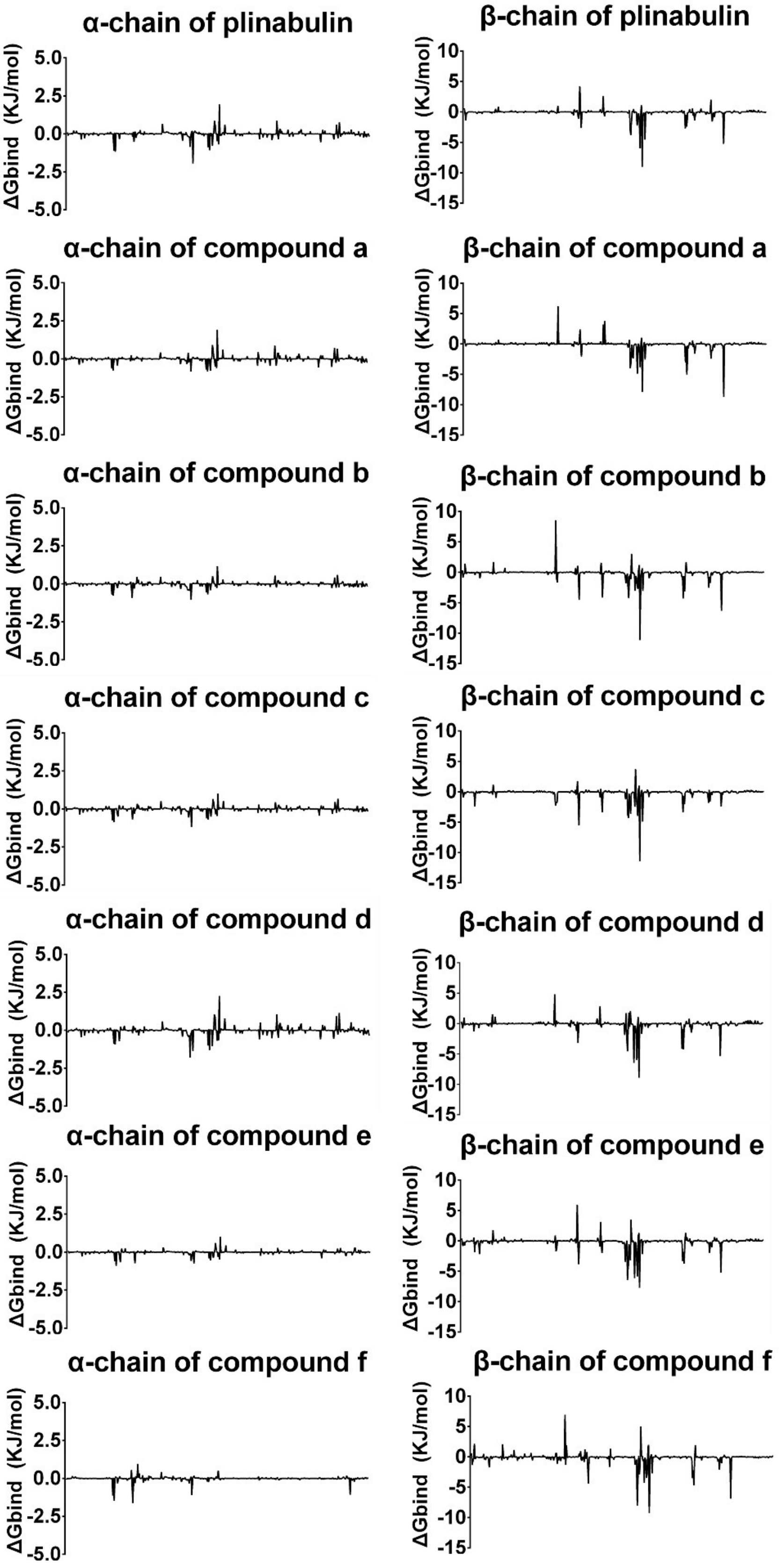

Fig. S1. Spectra of the inhibitor–residue interaction for the tubulin-DKP derivative complexes by α or β subunit.

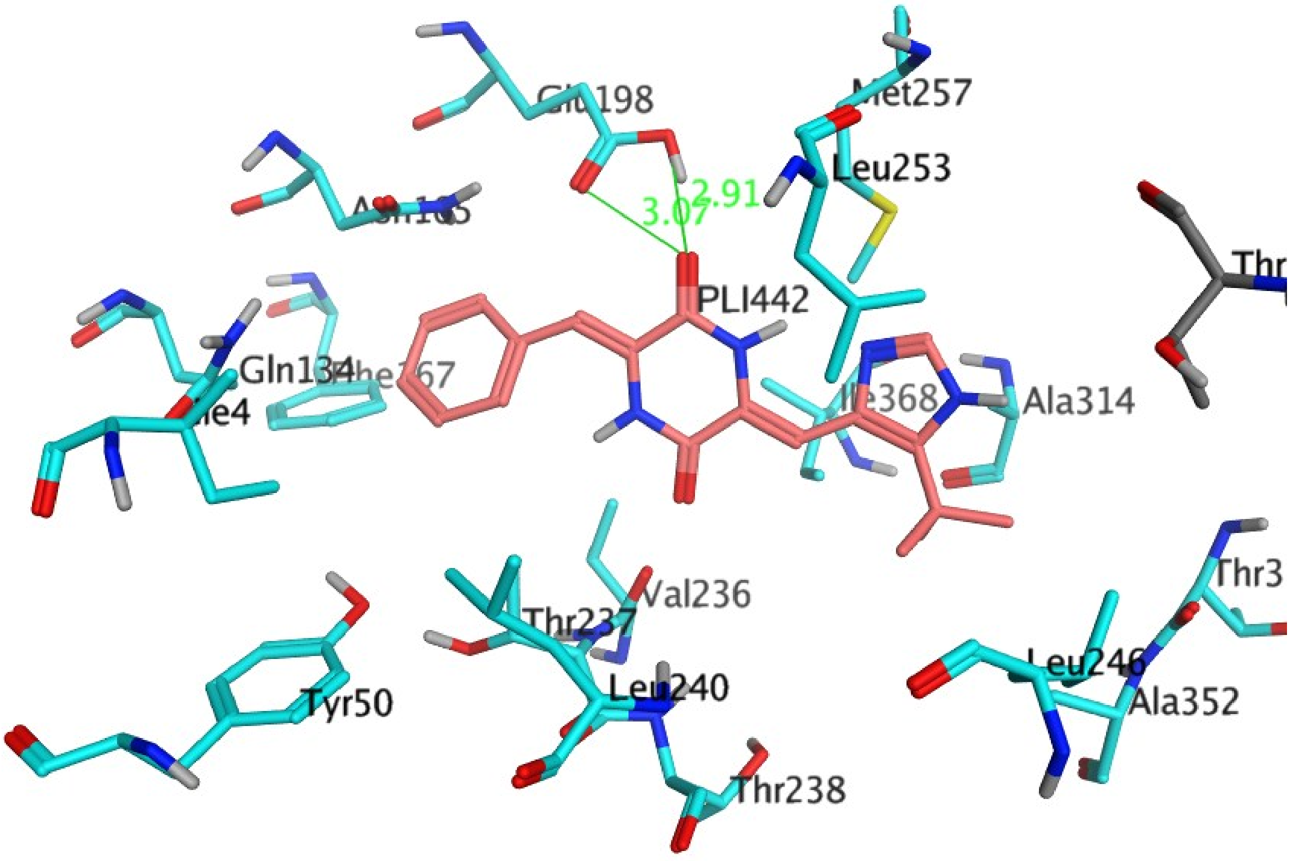

Fig. S2. Binding mode of plinabulin with tubulin.
